# A three-dimensional strain measurement method in elastic transparent materials using tomographic particle image velocimetry

**DOI:** 10.1371/journal.pone.0184782

**Published:** 2017-09-14

**Authors:** Azuma Takahashi, Sara Suzuki, Yusuke Aoyama, Mitsuo Umezu, Kiyotaka Iwasaki

**Affiliations:** 1 Department of Integrative Bioscience and Biomedical Engineering, Graduate School of Advanced Science and Engineering, Waseda University, Shinjuku, Tokyo, Japan; 2 Department of Modern Mechanical Engineering, Graduate School of Creative Science and Engineering, Waseda University, Shinjuku, Tokyo, Japan; 3 Cooperative Major in Advanced Biomedical Sciences, Graduate School of Advanced Science and Engineering, Waseda University, Shinjuku, Tokyo, Japan; Coastal Carolina University, UNITED STATES

## Abstract

**Background:**

The mechanical interaction between blood vessels and medical devices can induce strains in these vessels. Measuring and understanding these strains is necessary to identify the causes of vascular complications. This study develops a method to measure the three-dimensional (3D) distribution of strain using tomographic particle image velocimetry (Tomo-PIV) and compares the measurement accuracy with the gauge strain in tensile tests.

**Methods and findings:**

The test system for measuring 3D strain distribution consists of two cameras, a laser, a universal testing machine, an acrylic chamber with a glycerol water solution for adjusting the refractive index with the silicone, and dumbbell-shaped specimens mixed with fluorescent tracer particles. 3D images of the particles were reconstructed from 2D images using a multiplicative algebraic reconstruction technique (MART) and motion tracking enhancement. Distributions of the 3D displacements were calculated using a digital volume correlation. To evaluate the accuracy of the measurement method in terms of particle density and interrogation voxel size, the gauge strain and one of the two cameras for Tomo-PIV were used as a video-extensometer in the tensile test. The results show that the optimal particle density and interrogation voxel size are 0.014 particles per pixel and 40 × 40 × 40 voxels with a 75% overlap. The maximum measurement error was maintained at less than 2.5% in the 4-mm-wide region of the specimen.

**Conclusions:**

We successfully developed a method to experimentally measure 3D strain distribution in an elastic silicone material using Tomo-PIV and fluorescent particles. To the best of our knowledge, this is the first report that applies Tomo-PIV to investigate 3D strain measurements in elastic materials with large deformation and validates the measurement accuracy.

## Introduction

The mechanical interaction between blood vessels and medical devices can induce strains in these vessels. Measuring and understanding these strains is necessary to identify the causes of vascular complications [1–4]. Previous studies have relied on numerical structural analyses using the finite element method to evaluate the strain distribution in blood vessels [1–4]. However, the analysis conditions used were based on several assumptions that can significantly influence the results. This requires experimental validation [5–8]. Strain measurement techniques include strain gauging, digital image correlation, shearography, speckle interferometry, thermal stress analysis, and reflectometry. However, when using such techniques, the measured regions are limited to points or surfaces rather than three-dimensional (3D) regions inside the object [9]. This limitation implies that the results of numerical structural analyses are difficult to validate and highlights the challenges when measuring 3D strain distribution.

Digital volume correlation (DVC) allows for the generation of a 3D strain. In previous DVC studies, 3D images were obtained via X-ray computed tomography (CT) [9–13], magnetic resonance imaging (MRI) [14], and ultrasonic pulse-echo imaging [15, 16]. DVC has been used to measure strain in three dimensions in various materials, including bone [8, 9, 11–14] and skin tissues [15, 16]. However, measuring the strain in blood vessels using conventional DVC is impossible owing to the low time resolution of 3D imaging equipment such as CT, MRI, and ultrasonic pulse echo. When we target measurement of strain in blood vessels for example, the aorta has a typical inner diameter of 25 mm and a wall thickness of 2 mm that deforms at an average speed of 4.2 mm/s during a heartbeat.

Tomographic particle image velocimetry (Tomo-PIV) is used to measure the 3D flow velocity distribution at high spatial and time resolutions [17–23]. In Tomo-PIV, 2D images of flowing tracer particles in a fluid are captured using several cameras. The distribution of the displacements is then analyzed via a 3D reconstruction technique and DVC. In biomedical engineering applications, the flow velocity distribution is usually evaluated by tracking the flowing particle patterns in a transparent vessel model [22].

This study develops a method to measure 3D strain distributions using Tomo-PIV and compares the measurement accuracy of the strain between marked lines in tensile tests.

## Materials and methods

### Test specimen

**Fig 1:**
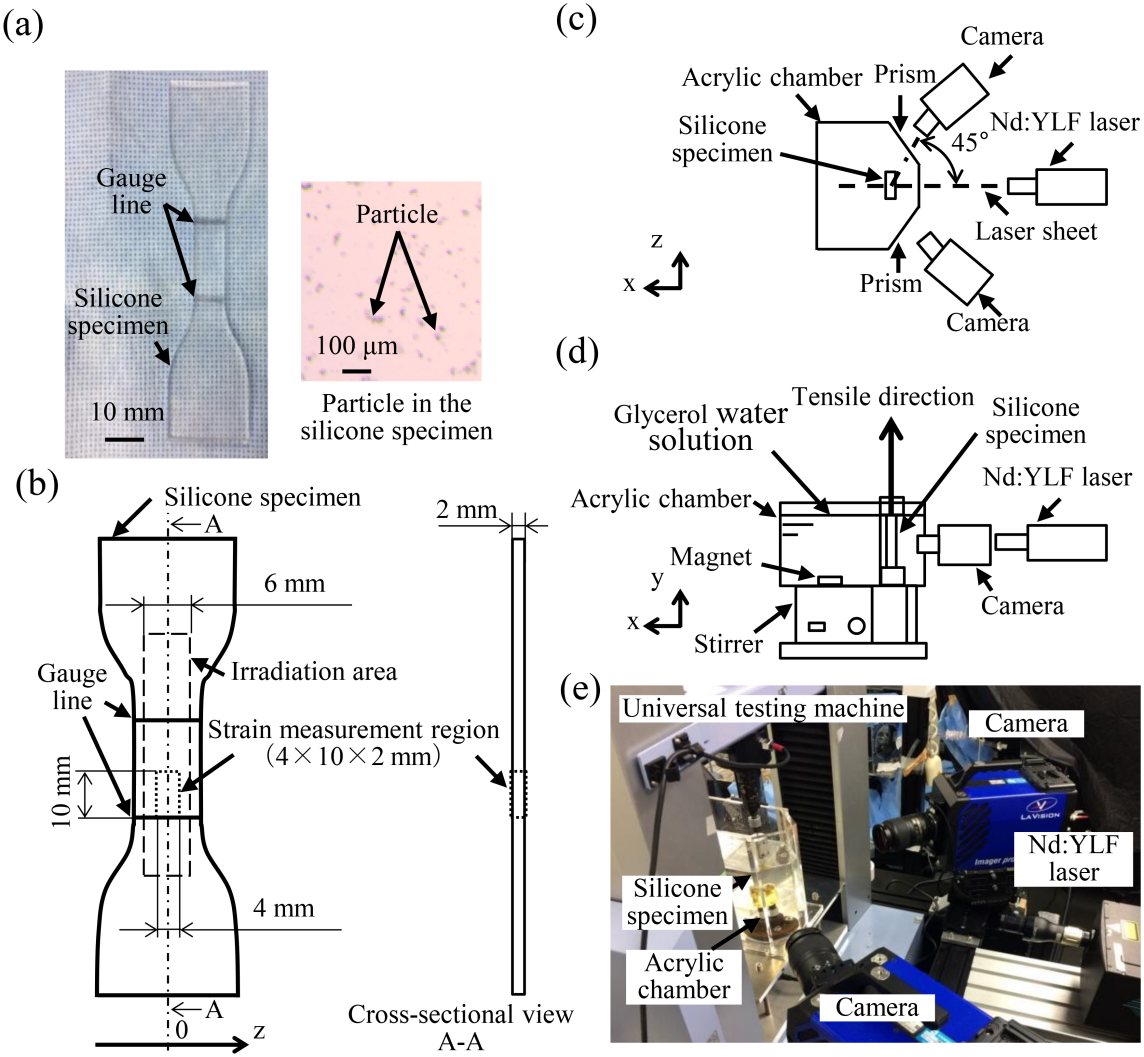
Schematic of the experimental system for measuring strain using Tomo-PIV. (a) Silicone specimen. (b) Schematic drawing of the area of strain measurement. (c) Top view of the experimental system. (d) Front view of the experimental system. (e) Experimental system.

The test specimens were made from transparent silicone (KE-1603, Shin-Etsu Chemical, Tokyo, Japan) mixed with fluorescent tracer particles with a mean diameter and density of 13 μm and 1.1 g/cm^3^, respectively (Fluostar, EBM, Tokyo, Japan). A planetary centrifugal mixer (AV-310, Thinky, Tokyo, Japan) was used to uniformly mix the particles and liquid silicone and deaerate the silicone. The liquid silicone was solidified via heating at 70°C for over 1 h in sheet form with a thickness of 2.00 ± 0.01 mm corresponding to the aorta wall thickness (Fig 1a). A silicone sheet with an elastic modulus of 2.9 ± 0.1 MPa was prepared. Then, type-2 dumbbell-shaped specimens were punched out according to Japanese Industrial Standards (JIS K 6251) and the International Organization for Standardization (ISO 37:2011). Two gauge lines were marked at 20-mm intervals along the parallel portion of the specimens (Fig 1a). Specimens with particle densities of 0.008, 0.014, 0.020, and 0.026 particles per pixel (ppp) were manufactured for this Tomo-PIV system to evaluate the influence of particle density on measurement accuracy.

### Experimental setup

The experimental setup consisted of two 12-bit complementary metal oxide semiconductor (CMOS) cameras with a full resolution of 2016 × 2016 pixels (Imager pro HS 4M, Lavision, Gottingen, Germany), a neodymium-doped yttrium lithium fluoride (Nd:YLF) laser (DS20-527, Photonics Industries, NY, USA), a universal testing machine (AG-X, Shimazu, Kyoto, Japan), an acrylic chamber, and a silicone specimen (Fig 1c, 1d and 1e). The experimental parameters are given in Table 1. Tensile tests were performed for the four specimens with different particle densities at a tensile speed of 50 mm/min [24, 25]. A 6-mm-thick laser sheet obtained using a beam expander and a series of optical lenses was irradiated at the center of the specimens (Fig 1b). The measurement region was 4 mm × 10 mm × 2 mm above the line of the lower gauge (Fig 1b). The cameras were angled at 45° along the direction of the laser sheet (Fig 1c). The camera lenses (Micro NIKKOR 85 mm, Nikon, Tokyo, Japan) had a focal distance of 85 mm and a lens aperture of f = 20. The cameras were focused on an area of approximately 45 mm × 45 mm, which corresponds to a magnification of M = 0.49. An aberration correction for the lens was performed using a calibration target (type 058–8, Lavision, Gottingen, Germany). Three iterative self-calibration corrections were used to reduce the calibration error to less than 0.3 pixels [23]. An acrylic chamber equipped with two viewing prisms was constructed to enable the orthogonal views of each camera. The acrylic chamber was filled with a glycerol water solution. The refractive index of the glycerol water solution was adjusted to that of the test specimen, 1.417 ± 0.002, to avoid refraction. To maintain the uniformity of the temperature and refractive index of the glycerol water solution, the solution was continuously mixed and maintained at 25°C using a thermal magnetic stirrer (MH301, Yamato Scientific, Tokyo, Japan).

**Table 1:** Experimental parameters for strain measurements.

Specimen	Material	Silicone (Refraction index n = 1.417)
	Particle	Fluorescent particle (Diameter: 13 μm, Density: 1.1 g/cm^3^)
	Particle density	0.008, 0.014, 0.020, 0.026 ppp
	Shape	Type-2 dumbbell shaped specimen (JIS K 6251, ISO 37:2011)
	Elastic modulus	2.9±0.1 MPa
Laser	Device	Nd:YLF laser
	Thickness	6.0 mm
Camera	Resolution	2016×2016 pixel
	Sampling rate	10 Hz
Image properties	Lens focal length	85 mm
	Lens aperture	f = 20
	Field of view	45×45 mm
	Image magnification	0.49
	Viewing angle	±iew
3D reconstruction	Method	MART (10 iterations), MTE (10 iterations)
	Resolution	22 μm/voxel
Digital volume correlation	Method	Iterative multi-grid volume deformation scheme
	interrogation voxel size	32×32×32, 40×40×40, 48×48×48, 56×56×56, 64×64×64 voxel
	Overlap	75%

### Measurement of displacement and strain

**Fig 2:**
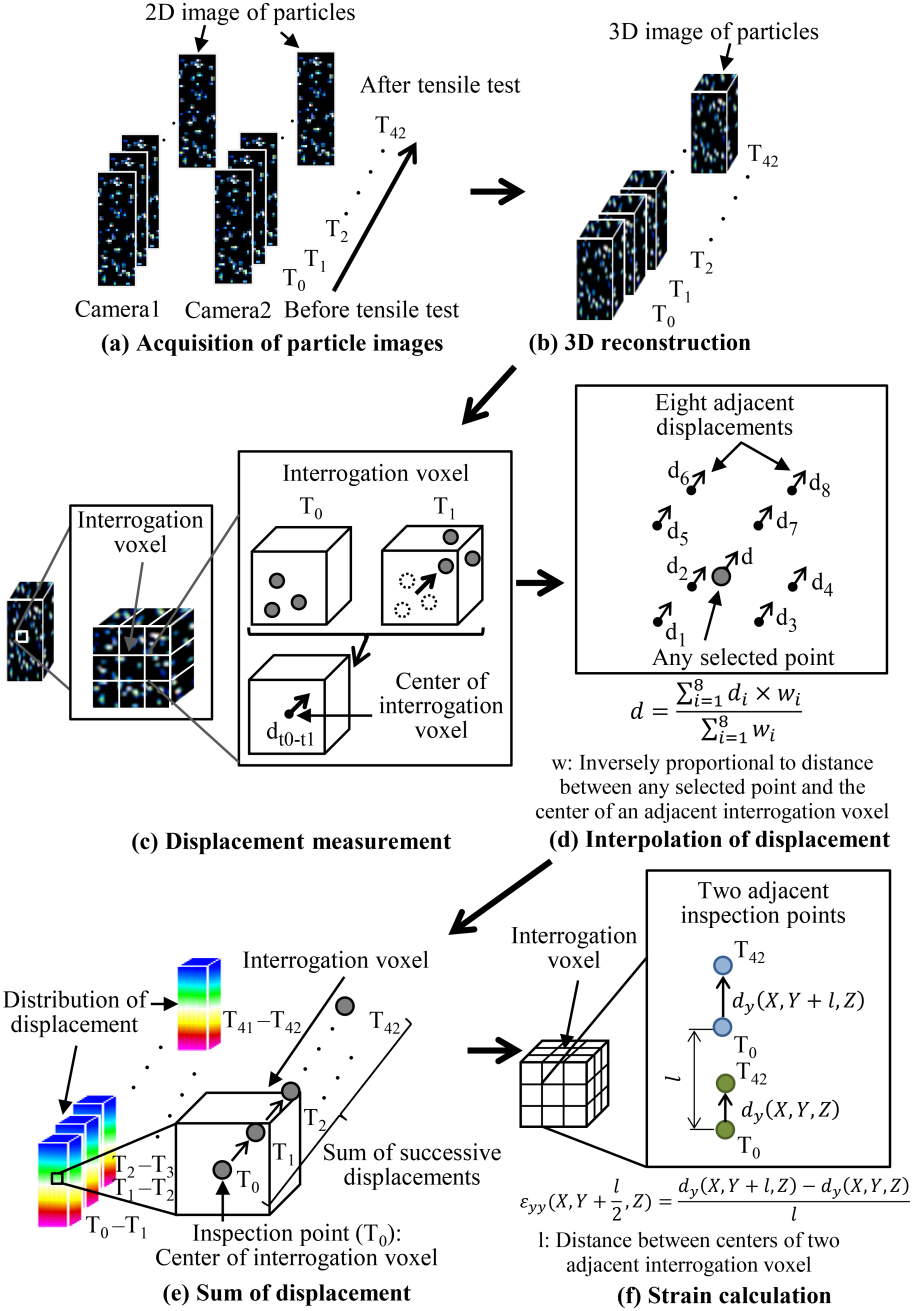
Procedure of 3D strain analysis in specimens incorporating fluorescent particles using Tomo-PIV.

The distribution of the normal strain in the tensile direction was calculated when the gauge strain was 10% using the following method. During the tensile test, 42 successive 2D images of the particles for each specimen were captured by the two cameras at a sampling rate of 10 Hz (Fig 2a), resulting in an acceptable average interframe particle displacement of 2–4 pixels in the measurement region [19, 22, 26]. The 3D images of the particles, with a discretization of 22 μm/voxel, were reconstructed from the 2D images using a multiplicative algebraic reconstruction technique (MART) [27] and motion tracking enhancement (MTE) with the number of objects set to N_o_ = 7 [18, 19] (Fig 2b). Ten iterations of the MART and MTE were conducted to improve the reconstruction accuracy [17, 18]. Each reconstructed 3D image was divided into cubic interrogation voxels (Fig 2c). Both the 3D reconstruction and the DVC were conducted using Davis 8.2.2 software (Lavision, Gottingen, Germany). The displacements of the center points of each interrogation voxel were calculated from two successive 3D images via a commonly used fast Fourier transform-based cross-correlation algorithm using an iterative multi-grid volume deformation scheme with a Gaussian-weighted volume [17, 20] (Fig 2c). The centers of each interrogation voxel at the starting point of the tensile test were defined as the inspection points. The displacement at any selected point was interpolated using eight adjacent displacements according to the following equation (Fig 2d):

$$d=\frac{\sum_{i=1}^{8}d_{i}\times w_{i}}{\sum_{i=1}^{8}w_{i}}.$$

Here, *d* is the displacement at each point and *w* is a weighting coefficient, which is inversely proportional to the distance between an inspection point and the center of an adjacent interrogation voxel. The displacements of each inspection point before and after the tensile test were estimated from the sum of their successive interpolated displacements during the tensile test (Fig 2e). The normal strain in the specimen in the tensile direction was calculated using the displacements of the inspection points before and after the tensile test according to the following equation (Fig 2f):

$$\varepsilon_{yy}\left(X,Y+\frac{l}{2},Z\right)=\frac{d_{y}\left(X,Y+l,Z\right)-d_{y}\left(X,Y,Z\right)}{l}$$

Here, *l* is the distance between the centers of two adjacent interrogation voxels and *d* is the displacement at each inspection point. Both the interpolated displacements and the distribution of the normal strain were computed in MATLAB (MathWorks, Massachusetts, USA).

### Influence of particle density and interrogation voxel size on measurement accuracy

The particle density and interrogation voxel size both influence the measurement accuracy. A high particle density in the measurement region allows for a higher spatial resolution. However, spurious particles (ghost particles), which lead to measurement errors, are generated at high particle density because the lines of sight to multiple particles intersect from several projections [20, 21]. Conversely, the number of particles in an interrogation voxel needs to be greater than at least 5–10 to perform a robust Tomo-PIV [9, 10, 17]. Interrogation voxel sizes of 24 × 24 × 24–64 × 64 × 64 voxels with a 50–75% overlap are typically adopted in Tomo-PIV to realize high spatial resolution with small interrogation voxel size and a sufficient number of particles in each interrogation voxel [17, 19, 22, 26]. Overlap indicates the overlap between neighboring interrogation voxels and is broadly used to measure local flow velocities [19, 22, 26]. The distance between the centers of two adjacent interrogation voxels was calculated according to the following equation:

$$\text{Distance between the centers of two adjacent interrogation voxels}=\text{interrogation voxel size }\times \;\frac{100-overlap\;ratio}{100}\;\times \;voxel\;discretization.$$

In this study, a 75% overlap was adopted in all tests to measure the local strain distribution.

#### (1) Influence of particle density

In terms of eliminating the measurement error due to the generation of ghost particles, the optimal particle density was investigated in a sufficiently large interrogation voxel size of 64 × 64 × 64 voxels. With reference to the reconstruction quality factor Q of Tomo-PIV using two cameras in a previous study [20], specimens with particle densities of 0.008, 0.014, 0.020, and 0.026 ppp were prepared. The average numbers of particles in the interrogation voxels in each specimen were 45, 91, 152, and 257, respectively, showing that the four specimens with different particle densities had sufficient numbers of particles to perform a robust Tomo-PIV.

#### (2) Influence of interrogation voxel size

To investigate the optimal interrogation voxel size in terms of having better spatial resolution, the normal strain distributions were compared between interrogation voxel sizes of 32 × 32 × 32, 40 × 40 × 40, 48 × 48 × 48, 56 × 56 × 56, and 64 × 64 × 64 voxels. The optimal density found in the previous experiment was used. A 75% overlap was applied for the measurement. The distances between the centers of two adjacent interrogation voxels for interrogation voxel sizes of 32 × 32 × 32, 40 × 40 × 40, 48 × 48 × 48, 56 × 56 × 56, and 64 × 64 × 64 voxels with a 75% overlap were 8, 10, 12, 14, and 16 voxels, respectively. The numbers of interrogation voxels used in the analyses were 14674, 7513, 4347, 2738, and 1834 voxels, respectively.

#### (3) Assessment of measurement conditions

The appropriate particle density and interrogation voxel size were examined in terms of measurement error, which was defined as the difference between the strain measured by Tomo-PIV and the gauge strain measured using binary images captured with one of the cameras of Tomo-PIV. The correlation value between two successive 3D images was used as an accuracy indicator of the displacement measurements under all conditions [10].

#### (4) Statistical analysis

In this study, a statistical analysis was performed using a software package (SPSS Statistics version21, IBM, NY, USA). For continuous variables, the distribution of normality and the equality of variances were assessed according to the Shapiro–Wilk and Levene tests, respectively [28, 29]. Tukey’s test was performed to provide multiple comparisons for the equality of normally distributed variables [29]. A P value lower than 0.05 was considered statistically significant.

## Results

### Influence of particle density

**Fig 3:**
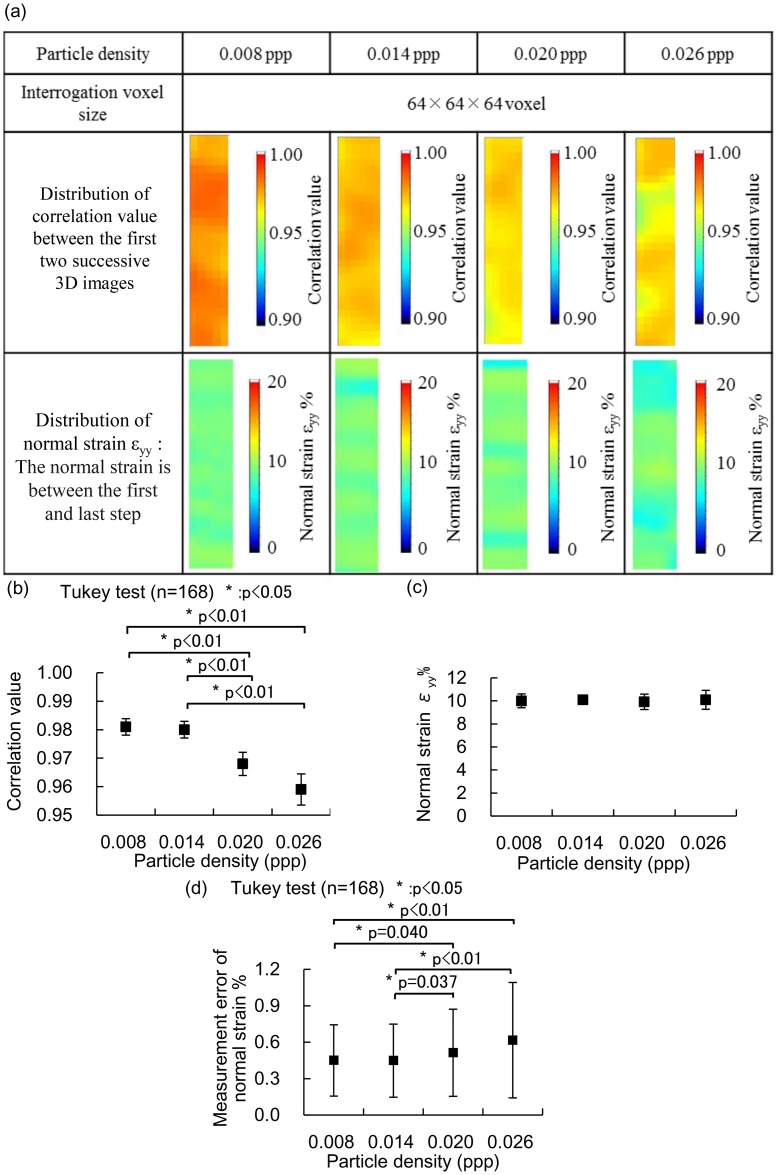
Influence of the particle density of specimens on 3D strain measurement using Tomo-PIV with interrogation voxel sizes of 64 × 64 × 64 voxels for the specimens. Specimens with particle densities of 0.008, 0.014, 0.020, and 0.026 ppp were compared at the cross section Z = 0 mm. (a) Distributions of the correlation values between the first two successive 3D images and the normal strain. (b) Correlation value between the first two successive 3D images. (c) Normal strain. (d) Measurement error of the normal strain.

The distributions of the correlation values and the normal strains in specimens with particle densities of 0.008, 0.014, 0.020, and 0.026 ppp at the cross section Z = 0 mm are shown in Fig 3a. The correlation value between the first two successive 3D images was higher than 0.94 over the entire measurement region of all the specimens (Fig 3a). The correlation values between other two successive 3D images showed equivalently high correlation values as that between the first two successive 3D images. The normal strains at the cross section Z = 0 mm were within 10 ± 2% for all the specimens. The mean correlation values for specimens with particle densities of 0.008 and 0.014 ppp were comparable, and those of 0.020 and 0.026 ppp decreased as particle density increased (Fig 3b). For all conditions, the mean normal strains were within 10 ± 0.1% (Fig 3c). The measurement errors of specimens with particle densities of 0.008 and 0.014 ppp were comparable and significantly smaller than those with particle densities of 0.020 and 0.026 ppp (Fig 3d). Based on these findings, the particle density of 0.014 ppp was selected in terms of its better measurement accuracy and spatial resolution.

### Influence of interrogation voxel size

**Fig 4:**
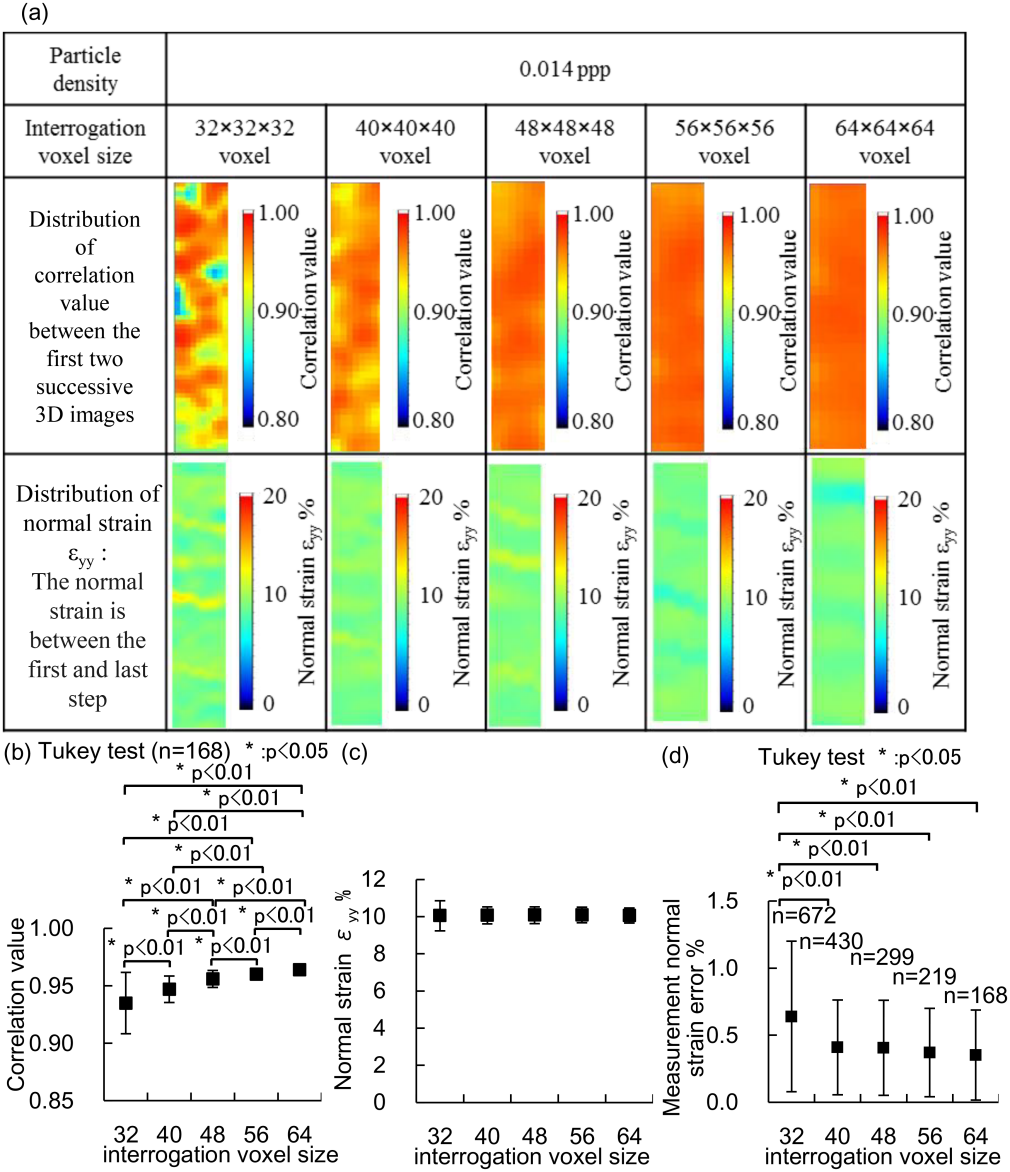
Influence of interrogation voxel size on the 3D strain measurements of specimens with a particle density of 0.014 ppp using Tomo-PIV. Interrogation voxel sizes of 32 × 32 × 32, 40 × 40 × 40, 48 × 48 × 48, 56 × 56 × 56, and 64 × 64 × 64 voxels for the specimen were compared at the cross section Z = 0 mm. (a) Distributions of the correlation values between the first two successive 3D images and the normal strain. (b) Correlation value between the first two successive 3D images. (c) Normal strain. (d) Measurement error of the normal strain.

The mean correlation values between the first two successive 3D images decreased as the interrogation voxel size decreased (Fig 4a and 4b). The mean correlation values between other two successive 3D images also decreased as the interrogation voxel size decreased. The normal strain measured with an interrogation voxel size of 32 × 32 × 32 voxels was locally higher than 13% (Fig 4a). The mean normal strains were 10 ± 0.1% for all interrogation voxel sizes (Fig 4c). The measurement error of the normal strain in an interrogation voxel size of 32 × 32 × 32 voxels was significantly higher than those in other interrogation voxel sizes (Fig 4d). The measurement errors were comparable for interrogation voxel sizes of 40 × 40 × 40, 48 × 48 × 48, 56 × 56 × 56, and 64 × 64 × 64 voxels (Fig 4d). Based on these data, the interrogation voxel size of 40 × 40 × 40 voxels was considered optimal in terms of its better measurement accuracy and spatial resolution.

**Fig 5:**
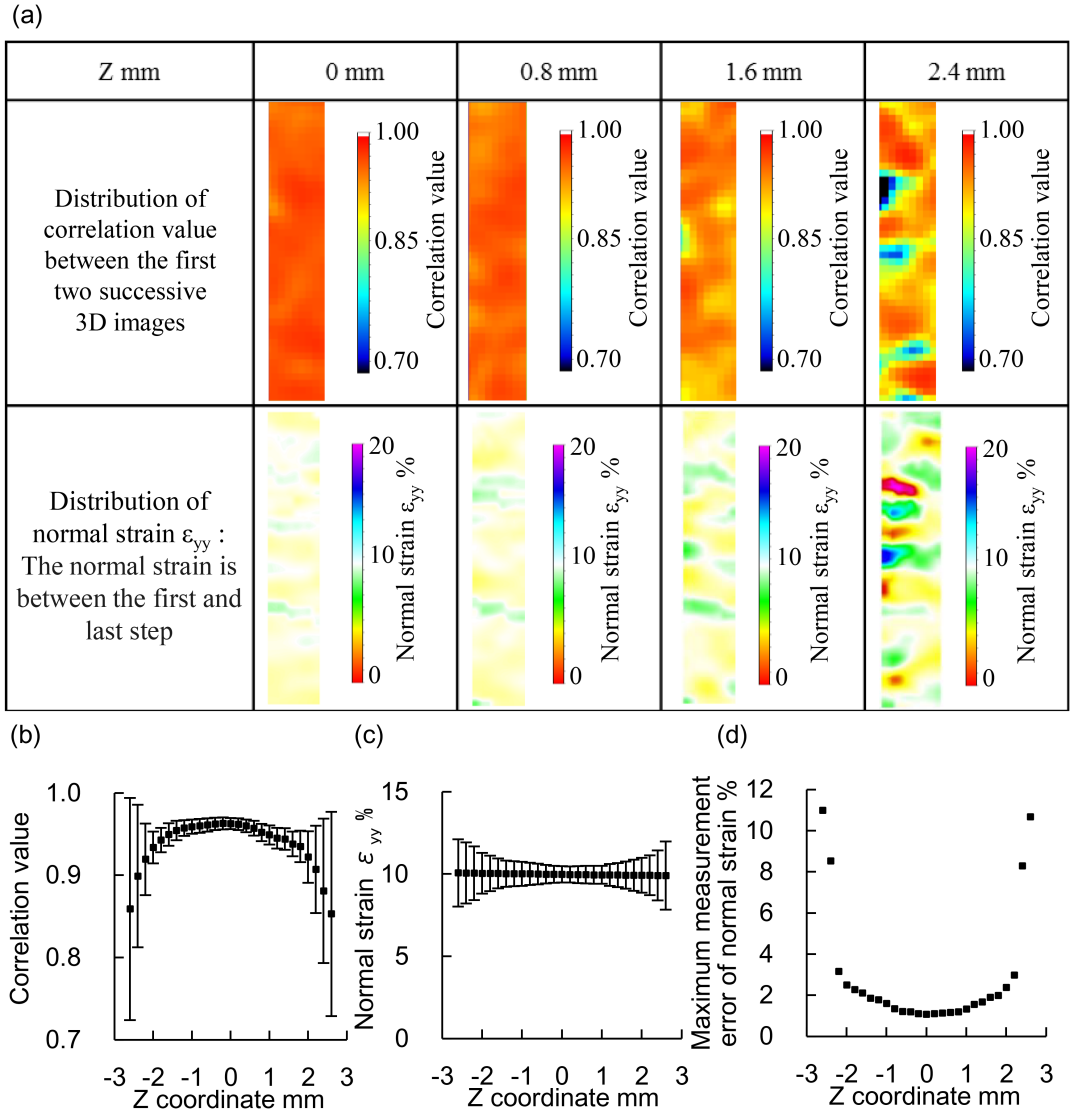
3D strain measurement using Tomo-PIV with an interrogation voxel size of 40 × 40 × 40 voxels for a specimen with a particle density of 0.014 ppp at each cross section. (a) Distributions of the correlation values between the first two successive 3D images and the normal strain. (b) Correlation value between the first two successive 3D images. (c) Normal strain. (d) Maximum measurement error of the normal strain.

The distributions of the correlation values between the first two successive 3D images and the normal strain in the cross sections of Z = 0, 0.8, 1.6, and 2.4 mm are shown in Fig 5a. The correlation value and the normal strain were locally low at the cross section at Z = 2.4 mm. The mean normal strains were 10 ± 0.1% at all cross sections (Fig 5c). The mean correlation value decreased and the maximum measurement error increased as the absolute value of Z increased owing to the low laser intensity at both surface layers of the 6-mm-thick laser sheet (Fig 5b and 5d). The maximum and minimum measurement errors of the normal strain in the measurement region between Z = −2 mm and Z = 2 mm were 2.5% and 1.1%, respectively.

## Discussion

In this study, we developed a method to experimentally measure strain distributions in elastic silicone materials using Tomo-PIV and fluorescent particles. The accuracy of the measurement method was evaluated in comparison to the gauge strain in the tensile test. The results show that the optimal particle density and interrogation window size in terms of the measurement accuracy and spatial resolution were 0.014 ppp and 40 × 40 × 40 voxels with a 75% overlap. Using this method, the distributions of the normal strain in elastic materials when the gauge strain was 10% were successfully quantified, and the mean normal strain in the specimen at the cross section Z = 0 mm was 10 ± 0.1%. The maximum measurement error of the normal strain was less than 2.5% in the measurement region in a 4-mm-wide region of the specimen. To the best of our knowledge, this is the first report that applies Tomo-PIV to investigate 3D strain measurements in elastic materials with large deformation and evaluates the measurement accuracy.

All previous studies using Tomo-PIV focused on flow investigations. Studies using DVC have shown that the measurement error of the displacement is between 0.01 and 0.1 voxels [10, 12, 24–28]. In the case where an interrogation size of 40 × 40 × 40 voxels with a 75% overlap was used in the measurement of the strain, the distance between the centers of two adjacent interrogation voxels in the tensile direction was 10 voxels. The displacements of the adjacent inspection points measured by the Tomo-PIV system included a measurement error between 0.01 and 0.1 voxels in each calculation using sequential 3D images. Therefore, the maximum measurement error in the distance between two adjacent inspection points was between 0.02 and 0.2 voxels, leading to a measurement error of the normal strain between 0.2% and 2%. In this study, the maximum measurement error of the normal strain was maintained at less than 2.5% in the 4-mm-wide region of the specimen even though the strain distribution was calculated from the sum of 41 successive interpolated displacements. These data indicate that the measurement system using Tomo-PIV presented here would be feasible for investigating 3D strain distributions in elastic materials with large deformation.

The accuracy of the strain measurement methodology presented here may be improved in the following way. In this study, we used two cameras in Tomo-PIV for the measurement of strain induced in the specimens, because the thickness of the specimens was 2 mm only. The measurement accuracy obtained in this study was satisfactory. However, the measurement using Tomo-PIV with two cameras has the limitations in the achievable seeding density. This can be overcome if three or more cameras are used in the imaging system. When a thicker specimen is tested, use of three or more cameras will contribute to the reduction of the intersection of lines of sight, and improvement of the measurement accuracy [17–23]. In an undeformed specimen, more accurate 3D reconstructions can be realized by acquiring images of the specimen from three or more different viewing directions using a single camera. In this study, the number of ghost particles was not measured because the optimal particle density could be determined based on measurement errors without counting the ghost particles. However, the accurate 3D distribution of particles in an undeformed specimen can be used to estimate the number of generated ghost particles for assessing the cause of the measurement error. Note that the evaluation of the 3D strain measurement was limited because the 3D strain was evaluated in comparison to the gauge strain, which is the average strain on the surface of the specimens. Nevertheless, a highly homogeneous strain was likely loaded in the specimen because the specimen was made from a single material with a uniform thickness of 2.00 ± 0.01 mm. Although, in this study, the displacements of the center points of each interrogation voxel were calculated from two successive 3D images via a commonly used fast Fourier transform-based cross-correlation algorithm [17, 20], application of direct correlation algorithm may further improve accuracy of the measurement presented here [30].

The method presented here will be useful to experimentally measure the 3D distribution of strain in elastic materials with large deformation. Accompanying the recent advancements in 3D modeling technology, the mechanical strain generated in cardiovascular tissues under external loads such as those caused by contact with medical devices can be quantified.

## Conclusions

We applied a novel method to measure the 3D strain yielded in an elastic silicone material using Tomo-PIV and fluorescent particles. In this study, the optimal particle density and interrogation voxel size were determined in terms of the measurement accuracy and spatial resolution. Distributions of the normal strain in elastic materials were successfully quantified. This study showed that the method presented here is feasible to accurately measure 3D strain distributions. The method may also be useful to understand strain distributions that occur in tissues such as blood vessels that are in contact with implantable medical devices.
